# Characterization of Proprotein Convertase Subtilisin/Kexin Type 9 (PCSK9) Trafficking Reveals a Novel Lysosomal Targeting Mechanism via Amyloid Precursor-like Protein 2 (APLP2)[Fn FN1]

**DOI:** 10.1074/jbc.M113.453373

**Published:** 2013-02-19

**Authors:** Rachel M. DeVay, David L. Shelton, Hong Liang

**Affiliations:** From Rinat-Pfizer Inc., South San Francisco, California 94080

**Keywords:** Cholesterol, Familial Hypercholesterolemia, Low Density Lipoprotein (LDL), Lysosomes, Protein Degradation, APLP2, APP, LDLR, PCSK9

## Abstract

Proprotein convertase subtilisin/kexin type 9 (PCSK9) regulates low density lipoprotein receptor protein levels by diverting it to lysosomes. Monoclonal antibody therapeutics aimed to neutralize PCSK9 have been shown to successfully lower serum LDL levels; however, we previously found that such therapeutic antibodies are subject to PCSK9-mediated clearance. In this study, we discovered that PCSK9 interacts via its C-terminal domain directly and in a pH-dependent manner with amyloid precursor protein as well as its closely related family member, amyloid precursor protein-like protein 2. Furthermore, we determined that amyloid precursor protein-like protein-2, but not amyloid precursor protein, is involved in mediating postendocytic delivery of PCSK9 to lysosomes and is therefore important for PCSK9 function. Based on our data, we propose a model for a lysosomal transport complex by which a soluble protein can target another protein for degradation from the luminal side of the membrane by bridging it to a lysosomally targeted transmembrane protein.

## Teaser

**Background:** PCSK9 functions to degrade the LDLR by a previously unknown lysosomal sorting mechanism.

**Results:** Here, we discovered and characterized a novel, pH-dependent interaction between PCSK9 and the amyloid precursor-like protein 2 (APLP2).

**Conclusion:** APLP2 plays an integral role in postendocytic PCSK9 lysosomal sorting.

**Significance:** These findings provide key insights into the mechanism by which PCSK9 degrades LDLR and reveal a novel lysosomal transport complex.

## Introduction

Cell surface receptors follow a variety of postendocytic trafficking routes that are generally mediated by sorting signals found in their cytosolic tails (1). Interestingly, alternate mechanisms exist to control transmembrane protein sorting. For example, the amyloid precursor-like protein 2 (APLP2),^2^ a type I transmembrane protein, alters the trafficking fate of MHC class I K^d^ molecules by directly binding and diverting them to lysosomes (2). Typically, however, unless a receptor has sorting signals present on its cytosolic tail, it is recycled back to the cell surface by default (3, 4). Because of its critical importance in regulating circulating LDL levels, the low density lipoprotein receptor (LDLR) has been studied exhaustively as an example of a recycled cell surface receptor.

LDLR is a multidomain receptor that is highly expressed in the liver and resides on the basolateral surface of hepatocytes. It comprises a large extracellular domain with seven ligand-binding repeats, three EGF repeat domains (EGF-A, EGF-B, and EGF-C), an *O*-linked sugar domain, and a YWTD domain (5). Following internalization, LDLR undergoes a conformational change upon exposure to the relatively acidic pH of endosomes that releases bound LDL. LDL is trafficked to lysosomes, whereas the receptor itself is recycled back to the cell surface (6). By this mechanism, LDLR removes LDL from the blood. High LDL serum levels correlate strongly with atherosclerosis and cardiovascular heart disease; thus, loss of function LDLR mutants were identified as determining factors of autosomal dominant familial hypercholesterolemia (5).

Another protein that has been causally linked to familial hypercholesterolemia is proprotein convertase subtilisin/kexin type 9 (PCSK9) (7–9). PCSK9 is a soluble protein that is secreted primarily from hepatic cells and that directly regulates serum LDL levels by targeting LDLR for lysosomal degradation (10, 11). In the endoplasmic reticulum, PCSK9 is autocatalytically cleaved, releasing its prodomain. The prodomain then reattaches near the catalytic domain and forces PCSK9 into an autoinhibitory conformation that lacks detectable protease activity (12). The catalytic domain of PCSK9 binds directly to the EGF-A domain of LDLR; indeed, key mutations (*e.g.* F379A) in this region block PCSK9/LDLR binding (13). After PCSK9 binds to LDLR on the cell surface, the complex is internalized, and their interaction tightens as pH levels drop along the endolysosomal route by which PCSK9 mediates LDLR degradation (13–16).

It is unknown how PCSK9 is targeted to lysosomes, but it is commonly presumed that PCSK9 binding to LDLR at the cell surface is required for PCSK9 endocytosis (16, 17). Indeed, a peptide mimicking a mutant form of LDLR EGF-AB domain blocks PCSK9 internalization presumably by blocking its interaction with LDLR (17). Interestingly, the lysosomal targeting and function of PCSK9 have been reported to rely on its C-terminal Cys-His-rich domain (CHRD), a region that is not required for PCSK9 interactions with LDLR (18–21). Thus, PCSK9 may interact with additional proteins to facilitate its trafficking. Indeed, Annexin A2 was shown to bind to the CHRD and in doing so inhibits PCSK9 function (22). The CHRD is subdivided into three repeat modules: M1, M2, and M3 (23). A recent study linking the CHRD to PCSK9 function focused on the roles of these modules and found that the M2 module plays a critical role in the extracellular pathway by which PCSK9 mediates degradation of its targets likely by sorting PCSK9 to lysosomes postendocytically (20).

We previously described a blocking antibody of PCSK9, J16, that completely disrupts PCSK9/LDLR interactions. J16 significantly increases LDLR levels while decreasing serum LDL levels in mice and non-human primates (24). Interestingly, during our studies, we observed that J16 exhibits PCSK9-mediated degradation *in vivo* (25). We therefore postulated that the PCSK9-antibody complex is internalized and trafficked to lysosomes via trafficking events that occur independently of direct PCSK9/LDLR interactions. In this study, we sought to better characterize PCSK9 trafficking and in doing so revealed that PCSK9 can divert LDLR to lysosomes from the luminal side of the membrane via a novel lysosomal transport complex.

## EXPERIMENTAL PROCEDURES

### 

#### 

##### Protein Expression and Purification

PCSK9, isotype control (IC) antibody, J10, and J16 were expressed and purified exactly as done before (24).

APLP2-extracellular domain (ECD) (amino acids 1–692) was cloned into pAcGFPN1 using the NheI/EcoRI sites (Clontech). The GFP tag was replaced with a His_6_ tag using AgeI/NotI sites, and a 3×FLAG tag was added using EcoRI/AgeI sites to make pAPLP2ECD. Amyloid precursor protein (APP)-ECD (amino acids 1–699) was PCR-amplified with a His_6_ tag on its C terminus and cloned into pAcGFPN1 using the NheI/SacII sites to make pAPPECD. PCSK9ΔCT was created by PCR amplification of PCSK9 residues 1–454 followed by a His_6_ tag. The PCR product was then cloned into pAcGFP using the EcoRI and BglII sites to make pPCSK9ΔCT. Expression vectors were transfected into HEK293 suspension cells. Supernatant from the transfected cells was collected after 5 days, filtered, dialyzed against PBS, and purified using cobalt beads (Thermo Scientific, Waltham, MA) according to the manufacturer's instructions. 5F6 antibody was identified in the same screen as described previously (24). It was purified from mouse ascites with protein A beads using standard techniques.

##### Cell Culture and siRNA Transfections

HepG2 cells were cultured in DMEM supplemented with 10% FBS, penicillin/streptomycin, and l-glutamine using standard tissue culture techniques. Huh7 cells were cultured identically. For siRNA knockdown, 20 μm siRNA oligos were transfected using RNAiMax Lipofectamine (Invitrogen) according to the manufacturer's instructions.

##### PCSK9 Treatment

PCSK9-mediated effects on LDLR or APLP2 were determined using methods similar to those described (24). Briefly, cells were switched into DMEM with 10% LPDS for at least 1 h prior to assay. 5 μg/ml PCSK9 was added to the cells in LPDS medium. After 6 h, cell lysates were harvested and loaded onto a 4–12% Bis-Tris gel (Invitrogen) before transferring to a nitrocellulose membrane for Western blot analysis. To assess the ability of 5F6 to block PCSK9-mediated LDLR degradation, 7.4 μg/ml (100 nm) PCSK9 was added to Huh7 cells in combination with increasing concentrations of 5F6 or a maximal concentration of IC as indicated in the figure. Lysates were harvested after 6 h before proceeding to Western blot analysis as described above. To assess PCSK9 sensitivity in siRNA-treated cells 72 h after siRNA transfection, cells were switched into DMEM with 10% LPDS for 1 h and then incubated with 50 μg/ml PCSK9 for 2.5 h to overcome possible thresholding effects or changes in transcription levels. Cell lysates were harvested before proceeding to Western blot analysis as described above.

##### Western Blot Analysis

Quantitative Western blotting was performed using standard techniques. Briefly, mouse anti-transferrin receptor (Invitrogen), goat anti-LDLR (R&D Systems, Minneapolis, MN), mouse anti-APP (Invitrogen), and rabbit anti-APLP2 (Abcam, Cambridge, MA) were used to decorate nitrocellulose membranes previously blocked using Odyssey blocking buffer (LI-COR Biotechnologies, Lincoln, NE). Secondary antibodies were donkey anti-mouse 680, goat anti-mouse 800, goat anti-rabbit 680, or donkey anti-goat 800 (LI-COR Biotechnologies). Proteins were detected with an Odyssey infrared detection system (LI-COR Biotechnologies). Integrated intensity signals were measured using Odyssey imaging software and normalized against the loading control transferrin receptor (TFNR).

The effects of PCSK9 on LDLR, APP, or APLP2 were determined by calculating their respective signals in PCSK9-treated cells as a percentage of their respective signals in untreated cells. The degree of LDLR degradation in siRNA-treated cells was calculated as percent LDLR signal in PCSK9-treated cells compared with untreated cells.

##### Internalization Assay

HepG2 cells were plated in a 96-well plate. PCSK9 was labeled with IRDye 800 according to the manufacturer's instructions (LI-COR Biotechnologies). 80 μm Dynasore or DMSO was added 30 min prior to the assay and kept in the medium throughout the experiment. The integrated intensity signal of PCSK9-IRDye was detected using an Odyssey infrared detection system, normalized to a general cell stain (Sapphire 700, LI-COR Biotechnologies), and plotted over time.

##### Quantitative PCR Analysis

Total RNA was isolated from HepG2 cells using an RNeasy extraction kit (Qiagen, Valencia, CA). cDNA was prepared using SuperScript II reverse transcriptase from 200 ng of RNA per reaction (Invitrogen). Quantitative PCR was performed in triplicate with TaqMan probes and TaqMan Gene Expression Master Mix according to the manufacturer's instructions using an Applied Biosystems StepOne Plus Real Time PCR system (Invitrogen) for three independent experiments. Samples were normalized against their respective RNA concentrations as described (26) and quantified as -fold change over negative control.

##### Trafficking Assays

PCSK9 or J16 was labeled with Alexa Fluor 488 or Alexa Fluor 647 according to the manufacturer's instructions (Invitrogen) with an average of two dye molecules per molecule.

For internalization assays, HepG2 cells were first plated on glass coverslips. Medium was exchanged for 10% LPDS medium 1 h prior to the assay. Assays were performed by premixing 5 μg/ml PCSK9 or PCSK9-488 with equimolar concentration of IC or J16. For 5F6 experiments, a 2.5-fold molar excess of antibody was used. The PCSK9-antibody complexes were added to cells for 4–6 h. Cells were then washed with PBS to remove surface-bound PCSK9, fixed with 4% formaldehyde for 10 min, and blocked with blocking buffer (2 mg/ml BSA and 10% donkey or goat serum, depending on the animal used to raise the secondary antibodies) before proceeding to immunofluorescence and confocal microscopy. Anti-APLP2 antibody trafficking was done the same way only using 4 μg/ml anti-APLP2 (R&D Systems). LDL-BODIPY or transferrin-488 (Invitrogen) internalization assays were done similarly except internalization was allowed for 1 h.

Surface localization was done in the same manner as internalization assays, but incubations were stopped after 1 h. Lysosomal trafficking or other subcellular localization analysis was also done in the same manner as internalization assays except after 4–6 h cells were washed, fixed, permeabilized with permeabilization buffer (0.1%Triton X-100, 2 mg/ml BSA, PBS, 0.02% Tween 20), and blocked before staining.

##### Immunofluorescence

Immunofluorescence staining was performed as described (25) following standard techniques. Primary antibodies used for immunofluorescence were mouse anti-Lamp2 antibody (Abcam), mouse anti-calnexin (Abcam), mouse anti-Golgin 97 (Abcam), goat anti-LDLR (R&D Systems), mouse anti-Lrp8 (Sigma), mouse anti-APLP2 (R&D Systems), and mouse anti-APP (Invitrogen). Isotype-specific secondary antibodies were all conjugated to Alexa Fluor dyes and were from Invitrogen. J10 and J16 were stained using secondary antibodies against mouse or human IgG, respectively (Invitrogen).

##### Microscope Image Acquisition and Analyses

Coverslips were mounted using ProLong Gold mounting medium (Invitrogen). Microscopy images from z stacks with 0.5-μm increments were collected using a 60×, 1.4 numerical aperture objective lens on a Leica laser-scanning confocal microscope at room temperature (Leica, Buffalo Grove, IL). Representative images from experiments are shown as projections of optical sections generated from Leica LAS AF software.

Internalized PCSK9-488 was determined by measuring the relative fluorescence intensity of PCSK9-488 in at least 100 cells per independent experiment using Leica LAS AF software. Relative intensities from LDLR siRNA-treated cells were calculated as a percentage of the average intensity of negative siRNA-treated cells. Lysosomal colocalization was determined by averaging the percent total PCSK9-488 or LDLR puncta associated with Lamp2 puncta. At least 500 puncta were counted for each of three independent experiments. The ImageJ colocalization Colormap plugin was used to further demonstrate the degree of lysosomal colocalization in APLP2 siRNA and 5F6 treatment experiments. The ImageJ plugin was used to calculate the colocalization correlation index (icorr) values where indicated.

##### Co-immunoprecipitations (Co-IPs)

HepG2 or HEK293 cells were grown to 80% confluence, harvested with Accutase, washed two times with PBS, and solubilized in IP buffer (3.5 ml/1 × 10^6^ cells; 0.5% Triton X-100, 20 mm Hepes, pH 6.0 or pH 7.4 as indicated), 150 mm NaCl, 20 mm CaCl_2_, 1× protease inhibitor mixture (Thermo Scientific) for 1 h at 4 °C. Cell lysates were then passed through a 27-gauge needle and cleared by ultracentrifugation (25,000 rpm for 30 min; SW-55ti rotor, Beckman-Coulter, Indianapolis, IN).

For co-IPs performed at pH 6.0 for mass spectrometry analysis, lysates were precleared by incubating with protein G Dynabeads (Invitrogen) for 30 min at 4 °C. PCSK9 (20 μg/ml) was premixed with J16 (40 μg/ml), added to the precleared lysates, and incubated for 2 h. J16 without PCSK9 was used as a negative control. The antibody-PCSK9 complex was pulled down using protein G beads, washed, and eluted with pH 8.0 IP buffer. Excess antibody was removed by incubation with protein A for 30 min. The remaining eluate was TCA-precipitated, resuspended, and loaded onto a 4–12% Bis-Tris gel (Invitrogen). The complex was run into the gel for 5 min without allowing for separation and stained with Coomassie, and the protein band was excised for LC-MS/MS analysis. Co-IPs performed at pH 7.4 for mass spectrometry were performed identically except using protein A Dynabeads (Invitrogen) and eluted by boiling.

For Western blot analysis, the co-IPs at both pH 6.0 and 7.4 were performed in the same manner using HepG2 cell lysates except using protein A beads instead and eluted by boiling in sample buffer. The complexes were run on 4–12% Bis-Tris NuPAGE gels (Invitrogen) and transferred to nitrocellulose membranes.

Co-IPs of purified, recombinant proteins were performed similarly using the same buffers. APLP2-ECD or APP-ECD was mixed with PCSK9 or PCSK9-ΔCΤ so the final concentration of all proteins was 3 μg/ml. J16 or IC was bound with protein A Dynabeads, excess antibody was removed, and 10 μl of IC- or J16-coated protein A beads was added to 500 μl of PCSK9/APLP2-ECD, PCSK9-ΔCT/APLP2-ECD, PCSK9/APP-ECD, or PCSK9-ΔCT/APP-ECD. Following a 30-min incubation, the beads were washed three times, and complexes were eluted by boiling in sample buffer. 5F6 co-IPs were performed exactly the same way but without APP-ECD or APLP2-ECD present.

##### Peptide Digestion and LC-MS/MS Analysis

Excised SDS-PAGE gel pieces were digested using a modified Shevchenko protocol and dried completely via vacuum centrifugation after extraction. Digested peptides were analyzed using a Paradigm MG4 HPLC system (Michrom Bioresources, Auburn, CA) joined with a Thermo Finnigan LTQ ion trap mass spectrometer (Thermo Fisher) using a Michrom Bioresources CaptiveSpray ionization source. Peptides were loaded onto a trap (Zorbax300SB-C18 (5 μm, 5 × 0.3 mm), Agilent Technologies, Santa Clara, CA) to be desalted and then eluted and separated using a reverse-phase Michrom Bioresources Magic C18AQ (200-μm × 150-mm) column at a flow rate of 2 μl/min. The gradient used for peptide elution included two main solvents, A and B (A, 0.1% formic acid; B, 100% acetonitrile). Specifically, the gradient was held at 2% B to 35% B for 80 min, raised to 80% B for 25 min, and held at 80% B for 1 min before decreasing to 2% B in 1 min. The column was then re-equilibrated for 13 min at 2% B and 98% A. The spray voltage was set to 1.8 kV with a heated transfer capillary temperature of 200 °C, and a full scan range of 350–1400 mass to charge ratio was used. The parameters for data-dependent MS/MS were as follows: 10 MS/MS spectra for the most intense ions from the full scan with 35% collision energy for collision-induced dissociation.

##### Criteria for Protein Identification from LC-MS/MS Analysis

Scaffold (version Scaffold_3_00_04, Proteome Software Inc., Portland, OR) was used to validate MS/MS-based peptide and protein identifications. Peptide identifications were accepted if they could be established at greater than 95.0% probability as specified by the PeptideProphet algorithm (27). Protein identifications were accepted if they could be established at greater than 99.0% probability and contained at least one identified peptide. Protein probabilities were assigned by the ProteinProphet algorithm (28). Only proteins identified in co-IPs from both HepG2 and HEK293 lysates were considered in subsequent analyses.

##### ELISAs

PCSK9/APLP2 ELISAs were carried out by coating MaxiSorp^TM^ plates (Thermo Scientific) with 5 μg/ml PCSK9 or 2% BSA. The plate was blocked by incubating with 2% BSA for 1 h at room temperature, washed five times using PBST (PBS + 0.05% Tween 20), and incubated with 4 μg/ml APLP2 for 2 h in Dulbecco's PBS (with calcium) or ELISA buffer (150 mm NaCl, 20 mm CaCl_2_, 20 mm MES buffer, pH 5.0, 5.5, 6.0, or 6.5). The plate was washed in corresponding pH buffer twice and fixed with 2% formaldehyde, 2% sucrose for 5 min. Plates were then washed 10 times with PBST. Bound APLP2 was identified using rabbit anti-APLP2 antibody (Abcam), a secondary anti-rabbit HRP (R&D Systems), and 3,3′,5,5′-tetramethylbenzidine (Kirkegaard & Perry Laboratories, Inc., Gaithersburg, MD) following standard ELISA protocols. APP/PCSK9 ELISAs were carried out the same way except plates were coated with APP and incubated with 1 μg/ml biotinylated PCSK9. Streptavidin-HRP and 3,3′,5,5′-tetramethylbenzidine were used to detect bound PCSK9. APP/PCSK9 and APLP2/PCSK9 ELISAs with 5F6 were carried out identically to this except using pH 6.0 buffer with increasing concentrations of 5F6 premixed with 1 μg/ml biotinylated PCSK9 as indicated in the figure.

APLP2/PCSK9/LDLR ELISAs were performed by coating MaxiSorp plates with 4 μg/ml APLP2. The indicated concentrations of PCSK9 were premixed with 2.5 μg/ml LDLR-ECD (R&D Systems). Complex inhibition ELISAs using IC, J16, or 5F6 were performed in the same manner except using 4 μg/ml PCSK9 premixed with 12 μg/ml IC, J16, or 5F6 in pH 6.0 buffer and added to APLP2-coated plates. Complexes were fixed as described above and detected using mouse anti-LDLR antibody (R&D Systems), goat anti-mouse HRP antibody (R&D Systems), and 3,3′,5,5′-tetramethylbenzidine.

##### Statistical Analyses

The ImageJ colocalization Colormap plugin was used to determine the icorr, which is the fraction of positively correlated pixels between two fluorescent populations, as described (29). The average with S.E. or S.D. was used for all analyses as indicated in the figure legends. Statistical significance was determined using a two-tailed, unpaired Student's *t* test for all analyses except PCSK9 or J16 internalization in siRNA-treated cells and the quantitative PCR analyses where statistical significance was determined using a two-tailed, paired Student's *t* test.

## RESULTS

### 

#### 

##### PCSK9 Follows Its Endolysosomal Route Regardless of a Direct LDLR Interaction

Previously, we reported an anti-PCSK9 blocking antibody, J16, that recognizes the LDLR binding epitope of PCSK9 and completely disrupts binding between PCSK9 and LDLR (24). To our surprise, this humanized antibody, J16, and its mouse precursor, J10, have dose-dependent half-lives in non-human primates and mice, respectively. The shortened half-lives of the antibodies at lower doses are dependent on PCSK9 (25). Similarly, other studies have reported short half-lives for PCSK9 antibodies that inhibit PCSK9/LDLR interactions (19, 30, 31). One explanation for this phenomenon is that PCSK9 follows its regular endolysosomal route regardless of a direct LDLR interaction and thereby leads to lysosomal degradation of PCSK9-bound antibodies.

**FIGURE 1.**
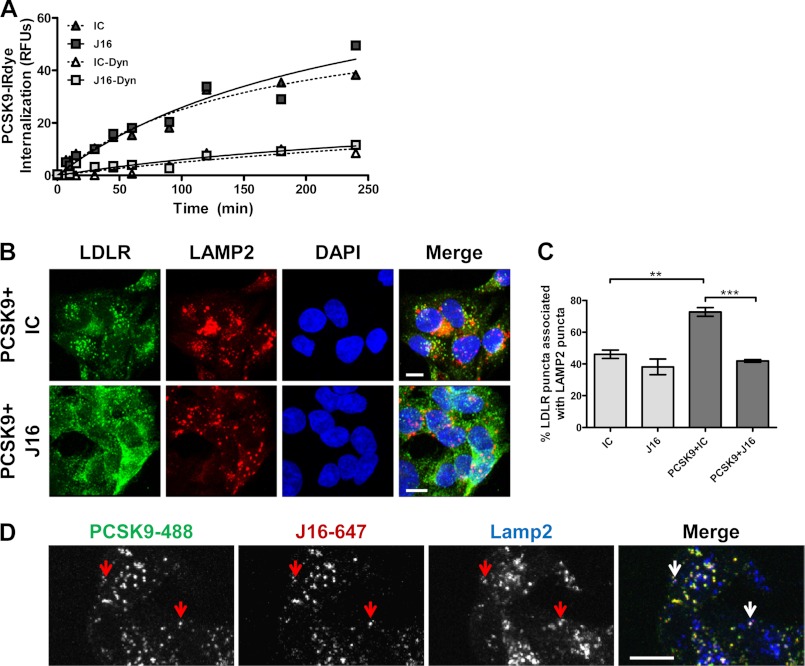
**Characterization of PCSK9 internalization and subsequent J16 or LDLR lysosomal trafficking in HepG2 cells.** *A*, internalization of PCSK9-IRDye in HepG2 cells plotted as the intensity of intracellular IRDye over time and normalized against a general cell stain. Assays were done in combination with J16 or IC in the absence or presence of the dynamin inhibitor Dynasore (*Dyn*) as indicated. *B*, colocalization of LDLR (*green*) with lysosomes (Lamp2; *red*) in DAPI (*blue*)-stained cells in the presence of PCSK9 + IC or PCSK9 + J16 as indicated. *Scale bars*, 10 μm. *C*, quantification of *B* as percentage of LDLR puncta associated with Lamp2 puncta. Shown is the average with S.E. (*error bars*) from three independent experiments. *D*, PCSK9-488 (*green*) and J16-647 (*red*) colocalization with Lamp2 (*blue*). *Arrows* indicate representative puncta showing colocalization among PCSK9-488, J16-647, and Lamp2. *Scale bars*, 10 μm. **, *p* < 0.005; ***, *p* < 0.0005.

To pursue this hypothesis, PCSK9 conjugated with an infrared dye (PCSK9-IRDye) was added to HepG2 cells in combination with J16 or an IC antibody, and PCSK9 internalization was determined at various time points with an infrared scanner. There were no significant differences in the overall accumulation or kinetics of PCSK9-IRDye signal in the presence of J16 as compared with IC (Fig. 1*A*), suggesting that PCSK9 does not require a direct LDLR interaction for internalization. Presence of the dynamin-specific inhibitor Dynasore inhibited the vast majority of PCSK9 internalization, indicating that PCSK9 internalization is dynamin-dependent and that the measured signal was specific to internalized, and not surface-bound, PCSK9-IRDye (Fig. 1*A*).

Because PCSK9 is endocytosed in the presence of J16, we postulated that J16 is internalized and routed to lysosomes via PCSK9 in place of LDLR and thereby allows LDLR to follow its default recycling route. To determine this, PCSK9 was added to HepG2 cells in combination with either J16 or IC, and LDLR colocalization with the lysosomal marker Lamp2 was then assessed by confocal microscopy. Consistent with previous reports, PCSK9 significantly enhanced LDLR lysosomal trafficking, whereas J16 reversed this effect (Fig. 1, *B* and *C*). Furthermore, exogenously added J16 directly labeled with Alexa Fluor 647 (J16-647) or complexed with PCSK9 labeled with Alexa Fluor 488 dye (PCSK9-488) was trafficked to lysosomes in HepG2 cells in accordance with its observed PCSK9-mediated degradation *in vivo* (Fig. 1*D*).

**FIGURE 2.**
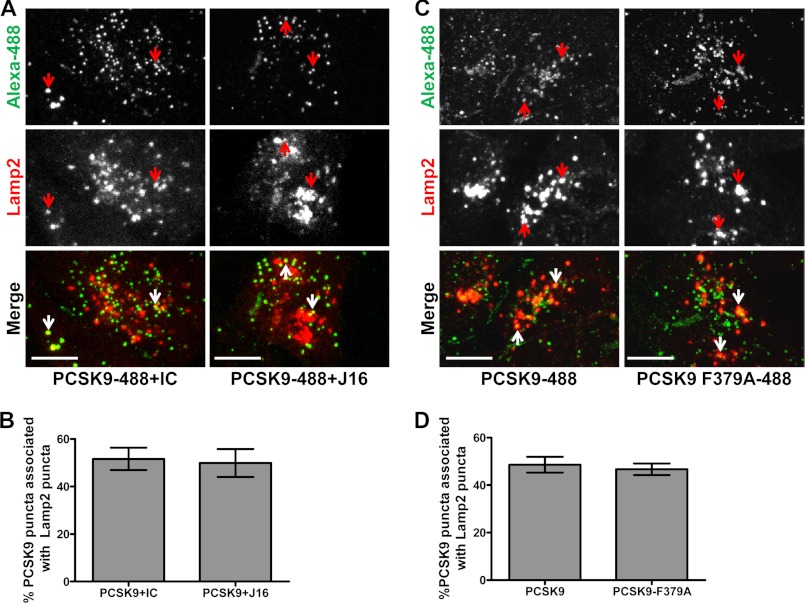
**PCSK9 lysosomal trafficking in the presence and absence of a direct LDLR interaction in HepG2 cells.** *A*, colocalization of PCSK9-488 (*green*) and lysosomal marker Lamp2 (*red*) in the presence of IC or J16 as indicated. *Arrows* indicate representative PCSK9-488 puncta associated with lysosomes. *Scale bars*, 10 μm. *B*, quantification of *A* expressed as the percentage of PCSK9-488 puncta colocalized with Lamp2 puncta. Shown is the average with S.E. (*error bars*) from three independent experiments. *C*, colocalization of PCSK9-488 or PCSK9-F379A-488 mutant (*green*) and lysosomal marker Lamp2 (*red*). *Arrows* indicate representative PCSK9-488 or PCSK9-F379A-488 puncta associated with lysosomes. *Scale bars*, 10 μm. *D*, quantification of *C* expressed as the percentage of PCSK9-488 or PCSK9-F379A-488 puncta colocalized with Lamp2-positive puncta. Shown is the average with S.E. (*error bars*) from three independent experiments.

As expected, exogenously added PCSK9-488 colocalized with lysosomes but not endoplasmic reticulum or Golgi (Fig. 2*A* and supplemental Fig. 1, A and B). Importantly, J16 did not have an effect on PCSK9 trafficking as there was no change in the percentage of PCSK9-488 puncta colocalized with Lamp2 when compared with IC (Fig. 2, *A* and *B*). In addition, the LDLR binding-deficient mutant PCSK9-F379A (13) was trafficked to lysosomes as efficiently as wild type PCSK9 following internalization (Fig. 2, *C* and *D*). Thus, although LDLR requires a direct interaction with PCSK9 to reach lysosomes, PCSK9 is internalized and routed to lysosomes regardless of its ability to interact with LDLR directly.

**FIGURE 3.**
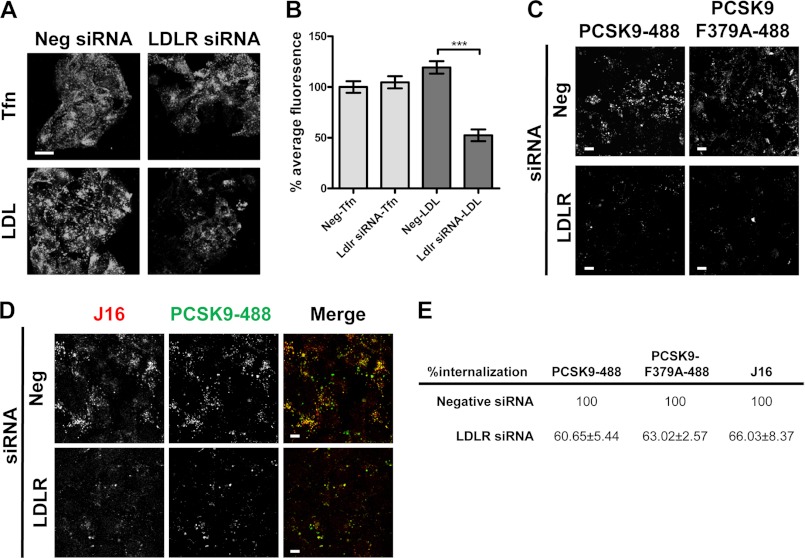
**Transferrin, LDL, and PCSK9-488 internalization in LDLR siRNA-treated HepG2 cells.** *A*, confocal microscopy of internalized transferrin-488 (*Tfn*) or LDL-BODIPY (*LDL*) in negative (*Neg*) or LDLR siRNA-treated HepG2 cells. *Scale bars*, 10 μm. *B*, quantification of *A* shown as the average percent intensity of negative siRNA cells with S.E. (*error bars*) from three independent experiments. *C–E*, internalized PCSK9-488 or PCSK9-F379A-488 (as indicated in *C*) or PCSK9-488/J16-647 (*D*) in negative control or LDLR siRNA-treated cells. *Scale bars*, 10 μm. Quantifications are shown in *E* as the average percent intensity of the negative control with S.E. from three independent experiments. ***, *p* < 0.0001.

Studies have shown that endocytosis of PCSK9 is dependent on the presence of LDLR (16, 17). To reconcile our findings that PCSK9 endolysosomal trafficking does not require a direct LDLR interaction, we knocked down LDLR using specific siRNA oligos and measured PCSK9 internalization. LDLR siRNA knocked LDLR protein levels down by 80% (supplemental Fig. 2A) and specifically blocked internalization of its ligand, LDL. Importantly, transferrin internalization was unaffected in these cells, indicating that general endocytosis was not disrupted by loss of LDLR (Fig. 3, *A* and *B*). In accordance with the literature, PCSK9 internalization was significantly blocked in LDLR siRNA-treated cells (Fig. 2, *C* and *E*). Surprisingly, however, PCSK9/J16 and PCSK9-F379A internalization were also attenuated in LDLR siRNA knockdown cells (Fig. 3, *C*, *D*, and *E*). Together, these data suggest that the presence of LDLR, but not a direct LDLR/PCSK9 interaction, is required for PCSK9 endocytosis.

##### PCSK9 Interacts Directly and in a pH-dependent Manner with APP and APLP2

We hypothesized that PCSK9 interacts with unknown protein(s) by epitopes that are distinct from that of J16/LDLR and that these interactions drive PCSK9 internalization and/or lysosomal targeting and subsequent degradation of PCSK9 targets. To identify novel binding partners of PCSK9, we performed co-IP experiments by adding J16-PCSK9 complexes to HepG2 or HEK293 cell lysates either at pH 7.4 to mimic the extracellular environment or at pH 6.0 to enrich for proteins likely to form a complex with PCSK9 in endosomes. The complexes were then analyzed by LC-MS/MS to identify co-immunoprecipitated proteins.

**FIGURE 4.**
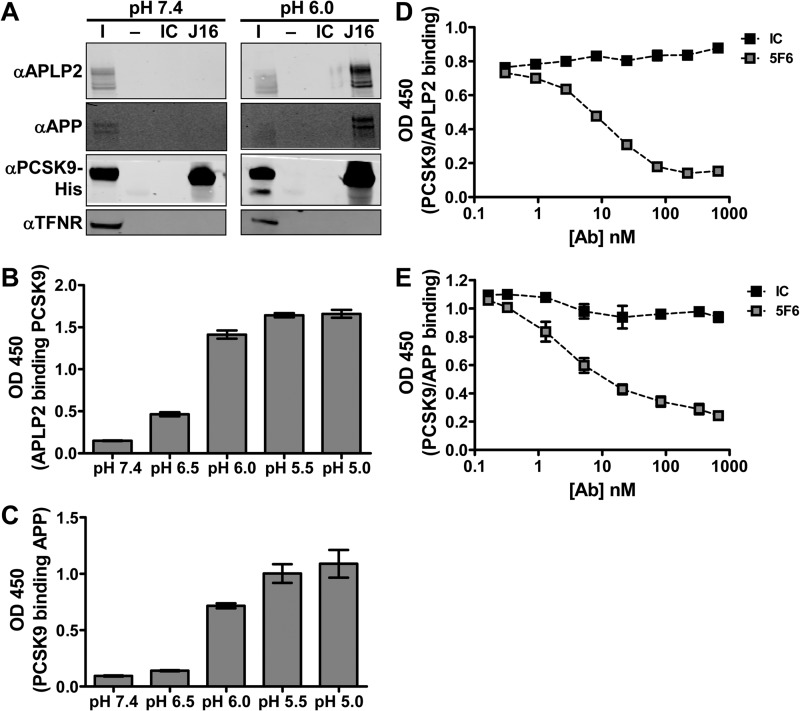
**Identification and biochemical characterization of interactions between PCSK9 and members of the amyloid precursor protein family.** *A*, Western blots showing APLP2, APP, PCSK9-His, or TFNR levels in input (*I*), IC IP, or J16 IP samples. co-IPs were performed at pH 6.0 or 7.4 using HepG2 lysates as described under “Experimental Procedures.” *B*, ELISA of APLP2 binding to PCSK9 at varying pH values. *C*, ELISA of biotinylated PCSK9 binding to APP at varying pH values. ELISA of biotinylated PCSK9 binding to APLP2-ECD (*D*) or APP-ECD (*E*) at pH 6.0 with increasing concentrations of 5F6 or IC. Shown is the average with S.D. (*error bars*) of duplicates from representative experiments.

As expected, PCSK9 was identified at both pH 7.4 and 6.0. Interestingly, the pH 6.0, but not the pH 7.4, complex consistently contained the APP as well as its closely related family member, APLP2, a protein that has been shown to deliver cell surface receptors to lysosomes from the plasma membrane (Table 1) (2). A Western blot of the co-IP confirmed that PCSK9 specifically interacts with multiple, large isoforms of APLP2 and APP in HepG2 whole cell lysate at pH 6.0 but not at pH 7.4 (Fig. 4*A*). Furthermore, both recombinant APLP2-ECD and APP-ECD showed pH-sensitive binding to PCSK9 by ELISA, indicating that PCSK9 binds directly to APLP2 and APP (Fig. 4, *B* and *C*). Interestingly, a monoclonal anti-PCSK9 antibody, 5F6, disrupts PCSK9 interactions with both APLP2-ECD (Fig. 4*D*) and APP-ECD (Fig. 4*E*). Thus, APLP2 and APP bind to a similar epitope on PCSK9 that can be specifically blocked using an anti-PCSK9 antibody.

**TABLE 1:** **List of proteins identified by mass spectrometry that co-immunoprecipitated with PCSK9 from HepG2 cells at pH 6.0** Proteins shown are present in samples co-immunoprecipitated from HepG2 cell lysates with 20 μg/ml PCSK9 and not present in those without PCSK9 using J16 at pH 6.0. These proteins met criteria explained under “Experimental Procedures” and were also present in a parallel co-IP experiment performed in Hek293 cells. Data shown are the number of unique peptides, the number of unique spectra, and the percent coverage of each protein. PCSK9, APP, and APLP2 are shown in bold. The only protein that met these criteria from the pH 7.4 co-immunoprecipitation was PCSK9.

Protein name	UniProtKB/Swiss-Prot accession number	No. unique peptides	No. unique spectra	Coverage
				%
**Proprotein convertase subtilisin/kexin type 9**	**sp Q8NBP7 PCSK9_HUMAN**	**17**	**25**	**28**
**Amyloid-like protein 2**	**sp Q06481 APLP2_HUMAN**	**16**	**26**	**29**
Ubiquitin C-terminal hydrolase 7	sp Q93009 UBP7_HUMAN	9	13	11
Uncharacterized protein C4orf14	sp Q8NC60 CD014_HUMAN	7	10	14
U5 small nuclear ribonucleoprotein 200-kDa helicase	sp O75643 U520_HUMAN	7	10	3.4
**Amyloid β A4 protein**	**sp P05067 A4_HUMAN**	**5**	**6**	**8.1**
Transcription elongation factor SPT6	sp Q7KZ85 SPT6H_HUMAN	4	6	5.2
Inositol 1,4,5-trisphosphate receptor type 3	sp Q14573 ITPR3_HUMAN	3	3	0.75
Coatomer subunit α	sp P53621 COPA_HUMAN	2	2	2
Squamous cell carcinoma antigen recognized by T-cells 3	sp Q15020 SART3_HUMAN	2	6	3
Protein transport protein Sec23A	sp Q15436 SC23A_HUMAN	2	2	3.9
Peptidyl-prolyl cis-trans isomerase-like 4	sp Q8WUA2 PPIL4_HUMAN	1	1	3.5
Sorting nexin-9	sp Q9Y5X1 SNX9_HUMAN	1	1	2.4

##### APLP2 and APP Bind to the CHRD of PCSK9

**FIGURE 5.**
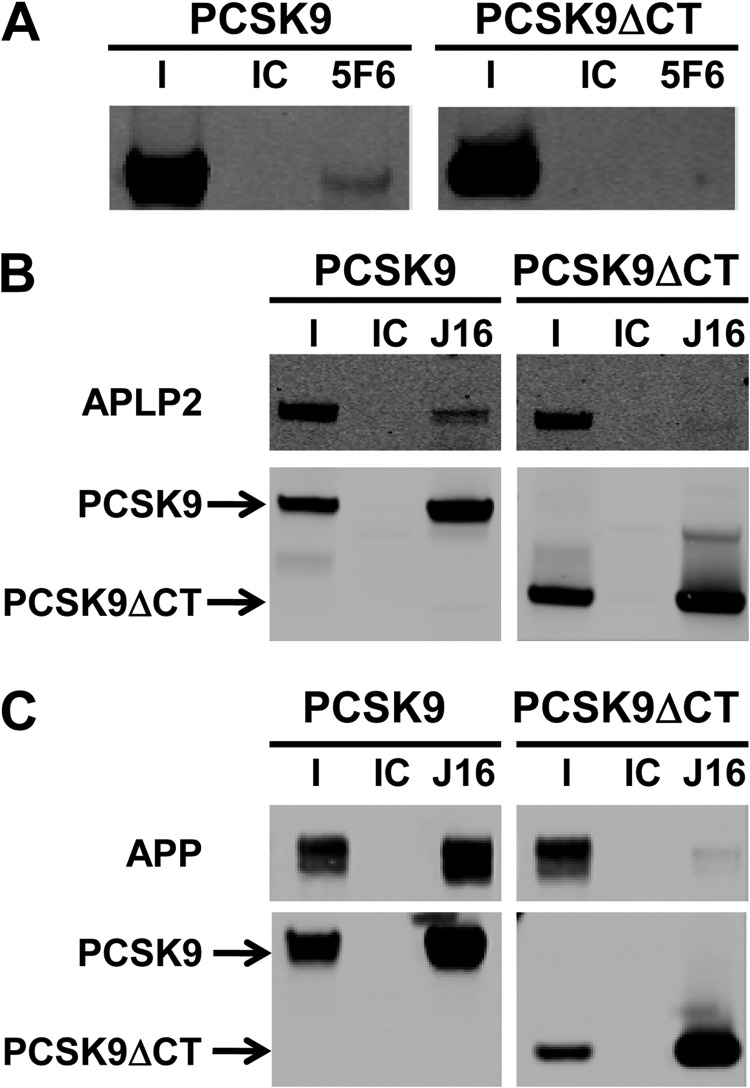
**Role of the CHRD of PCSK9 in APP and APLP2 interactions.** *A*, Western blot of immunoprecipitation of PCSK9 or PCSK9ΔCT using IC or 5F6 as indicated. *I* is the input fraction; *IC* and *5F6* indicate isotype control- or 5F6-immunoprecipitated fractions, respectively. *B*, Western blot of co-immunoprecipitation experiment in which APLP2-ECD was combined with PCSK9 or PCSK9ΔCT and immunoprecipitated using IC or J16. *I* is the input fraction; *IC* and *J16* indicate isotype control- or J16-immunoprecipitated fractions, respectively. Membranes were probed for APLP2 or PCSK9-His as indicated. *C*, same as *B* but using APP-ECD. Membranes were probed for APP-ECD or PCSK9-His as indicated.

The CHRD at the C-terminal region of PCSK9 is not required for PCSK9/LDLR binding, but it has been implicated in PCSK9 trafficking and function presumably through interactions with unidentified partners. To determine whether the CHRD is required for APLP2 and APP binding to PCSK9, we tested the ability of 5F6 to immunoprecipitate either full-length PCSK9 or a recombinant, C-terminal truncation of PCSK9 lacking the CHRD (PCSK9ΔCT). 5F6 successfully immunoprecipitated recombinant full-length PCSK9, but interestingly, it did not interact with PCSK9ΔCT (Fig. 5*A*).

To test more directly whether the CHRD is required for PCSK9 interactions with APLP2 or APP, recombinant APLP2-ECD or APP-ECD was incubated with full-length PCSK9 or PCSK9ΔCΤ and immunoprecipitated using J16. Consistent with co-immunoprecipitations from cell lysates, full-length PCSK9 was immunoprecipitated by J16 but not IC and was complexed with APLP2-ECD (Fig. 5*B*) or APP-ECD (Fig. 5*C*) at pH 6.0. However, although PCSK9ΔCT was specifically immunoprecipitated by J16, it did not interact with APLP2-ECD or APP-ECD (Fig. 5, *B* and *C*). Together, these data suggest that PCSK9 binds directly to APLP2 and APP via the CHRD.

##### PCSK9-mediated LDLR Degradation in HepG2 Cells Is Dependent on APLP2 but Not APP

**FIGURE 6.**
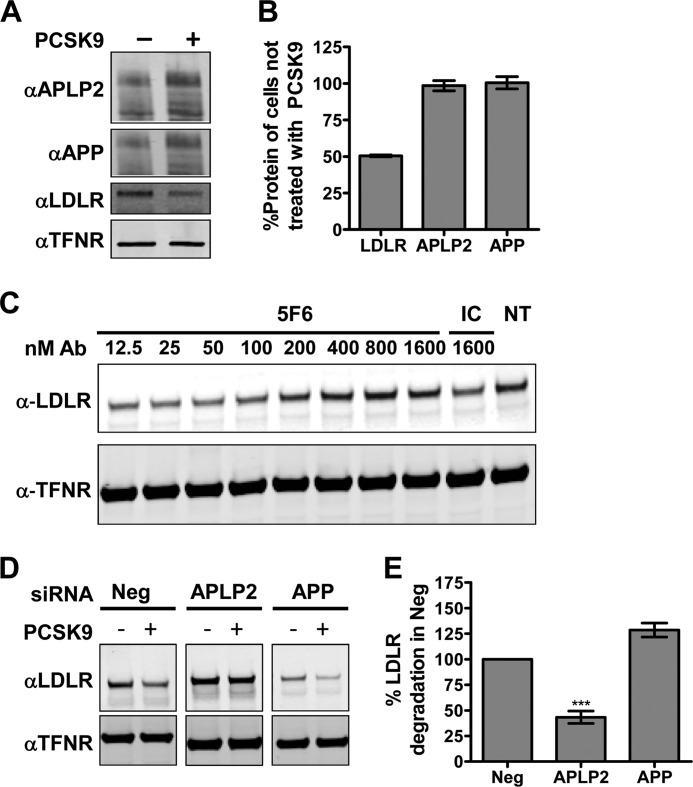
**Roles of APP or APLP2 in PCSK9 function.** *A*, Western blot of total APP, APLP2, LDLR, and TFNR in HepG2 cells incubated with or without 5 μg/ml PCSK9. *B*, quantification of *A*. LDLR signals were normalized to those of TFNR and are shown as the average percent untreated signal with S.E. (*error bars*) from three independent experiments. *C*, Western blots of LDLR and TFNR from Huh7 cells treated with 7.4 μg/ml (100 nm) PCSK9 combined with IC or increasing concentrations of 5F6 as compared with non-treated (*NT*) cells. *D*, Western blot of LDLR or TFNR from HepG2 cells treated with negative (*Neg*) control siRNA, APLP2 siRNA, or APP siRNA as indicated and incubated for 2.5 h with 50 μg/ml PCSK9 as indicated. Samples were processed on the same gel and membrane with cropped images presented. *E*, quantification of *D*. LDLR signals were normalized to TFNR and are shown as the average percentage of LDLR degradation normalized against negative control siRNA-treated cells with S.E. (*error bars*) from three independent experiments. *Ab*, antibody. ***, *p* = 0.003.

PCSK9 has been shown to bind multiple cell surface receptors and subsequently target them for lysosomal degradation (32, 33). To understand whether APLP2 and APP are novel targets of PCSK9, we treated HepG2 cells with exogenous PCSK9. As expected, addition of PCSK9 lowered total LDLR protein levels by 50%. In contrast, neither APLP2 nor APP protein levels were altered (Fig. 6, *A* and *B*).

Consistent with reports that the CHRD is important for PCSK9 function, 5F6 blocked PCSK9-mediated LDLR degradation in Huh7 cells in a dose-dependent manner (Fig. 6*C*). Thus, APLP2 and/or APP is likely involved in PCSK9 function. To dissect the individual roles of APLP2 and APP, the two genes were knocked down in HepG2 cells using specific siRNA oligos. APLP2 and APP protein levels were successfully lowered to an average of 11 and 17%, respectively, of that detected in negative control siRNA cells (supplemental Fig. 2, B and C). Interestingly, both APLP2 and APP siRNA treatment had significant and opposite (33 and −46%, respectively) effects on LDLR transcription levels after 72 h, likely accounting for the differences in LDLR protein levels in PBS-treated samples (supplemental Fig. 2D). Consistent with these results, APLP2 knockdown increased surface-exposed LDLR and subsequently enhanced LDL internalization, whereas APP showed the opposite effect (supplemental Fig. 3, A and B).

When APP siRNA-treated cells were incubated with PCSK9, a modest increase in the percentage of LDLR degraded relative to negative siRNA control cells (Fig. 6, *D* and *E*) was observed, suggesting that APP is not required for PCSK9-mediated LDLR degradation. In contrast, APLP2 siRNA-treated cells were significantly protected from PCSK9 treatment as evidenced by a 55% reduction in the percentage of LDLR degraded compared with negative control cells (Fig. 6, *D* and *E*), indicating that APLP2 plays an important role in PCSK9 function.

##### PCSK9 and LDLR Are Spatially Localized to the Same Regions on the Cell Surface as APLP2 but not APP

Because the CHRD has been shown to play some, albeit largely unknown, role in PCSK9 trafficking, we wanted to study the endolysosomal route of PCSK9 relative to APP or APLP2. Importantly, we did not observe appreciable direct APLP2 or APP interactions with PCSK9 at neutral pH under the conditions tested. Thus, for either of these proteins to efficiently and directly modulate postendocytic PCSK9 sorting, we hypothesized that they would need to be coordinately endocytosed to enable complex formation upon exposure to endosomal pH. Prior to endocytosis, cell surface proteins cluster on the cell surface (34). We therefore looked at PCSK9 localization relative to LDLR-, APLP2-, or APP-rich clusters on the cell surface as a measure of whether these proteins are internalized together.

Confocal microscopy revealed that PCSK9 conjugated with Alexa Fluor 647 (PCSK9-647) colocalized strongly with cell surface LDLR clusters (55%) in contrast to its colocalization with surface Lrp8 (20%), another known PCSK9 target (supplemental Fig. 4, A, B, and C) (32, 33, 35). Interestingly, PCSK9-647 colocalized with proportionately the same number of LDLR-positive surface clusters when their direct binding was disrupted by the presence of J10. As expected, J10 was also colocalized with PCSK9/LDLR-positive puncta (supplemental Fig. 4, B and C).

**FIGURE 7.**
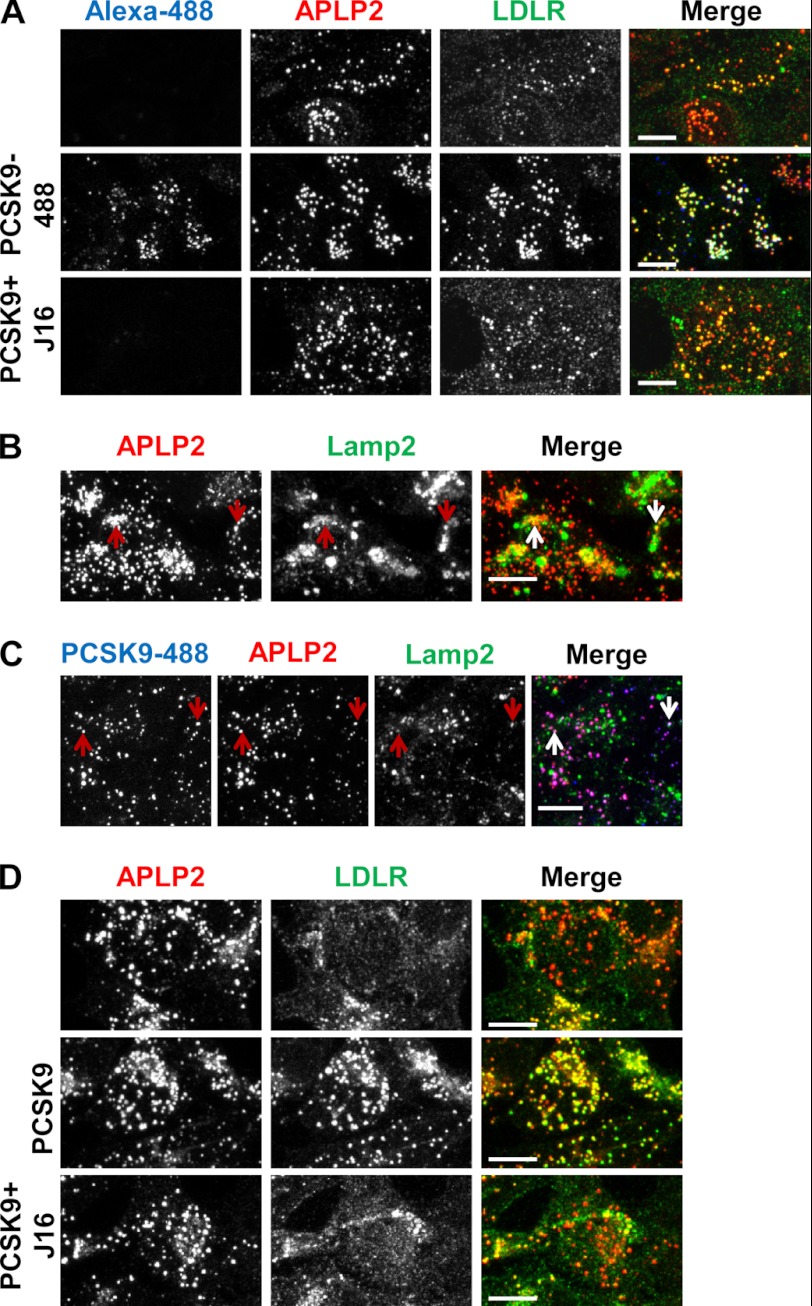
**Characterization of the endocytic route of APLP2 and colocalization with PCSK9 and LDLR.** *A*, APLP2 (*red*) and LDLR (*green*) surface staining on HepG2 cells in the absence (*top*), or presence of PCSK9–488 (*middle, blue*), or presence of PCSK9/J16 (*bottom*). *B*, colocalization of internalized anti-APLP2 monoclonal antibody (*red*) with Lamp2 (*green*). Examples are indicated by *arrows. C*, colocalization of internalized anti-APLP2 monoclonal antibody (*red*) with internalized PCSK9-488 (*blue*) and with Lamp2 (*green*). Examples are indicated by *arrows. D*, APLP2 (*red*) colocalization with LDLR (*green*) with or without exogenously added PCSK9 or PCSK9/J16 as indicated. *Scale bars*, 10 μm.

In addition to surface LDLR clusters, we observed that exogenously added PCSK9-488 was also highly colocalized with surface APLP2 clusters with an average colocalization icorr value of 0.65 ± 0.08. Significantly, LDLR clusters on the cell surface were strongly colocalized with APLP2 cell surface clusters with an average icorr value of 0.83 ± 0.20 when in the presence of PCSK9 (Fig. 7*A*). Together, these results suggest that PCSK9 and LDLR are spatially distributed on the cell surface with APLP2 and indicate that the three proteins may be internalized in the same endocytic compartments. Interestingly, cells incubated with J16/PCSK9 or cells without exogenously added PCSK9 still showed significant LDLR/APLP2 surface colocalization albeit to a lesser degree compared with when PCSK9 was present, indicating that LDLR and APLP2 cluster to the same regions on the cell surface and therefore may normally be endocytosed together (Fig. 7*A*).

**FIGURE 8.**
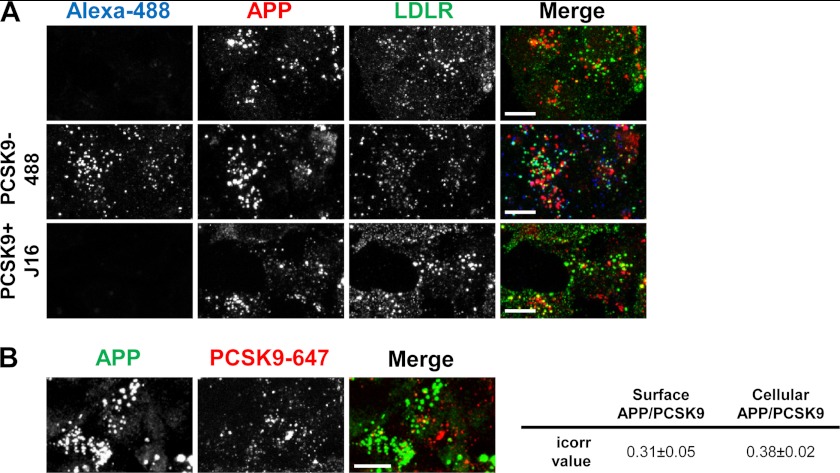
**APP surface and cellular localization relative to LDLR or PCSK9.** *A*, APP (*red*) and LDLR (*green*) surface staining on HepG2 cells in the absence (*top*), or presence of PCSK9–488 (*middle; blue*), or presence of PCSK9/J16 (*bottom*). *B*, APP (*green*) and internalized PCSK9-647 (*red*) in HepG2 cells. icorr values of cell surface or total cellular APP/PCSK9 colocalization are shown in the table on the *right. Scale bars*, 10 μm.

In further support of APP not directly mediating PCSK9 trafficking and function, we did not observe a high degree of colocalization between PCSK9 and APP on the cell surface (Fig. 8*A*). Moreover, APP colocalization with LDLR was also relatively minimal, and addition of PCSK9 did not visibly enhance their colocalization (Fig. 8*A*). Based on these data, it is likely that APP is not normally endocytosed in the same compartments with PCSK9 and/or LDLR.

##### PCSK9 Follows the Same Endolysosomal Route as APLP2

Consistent with previous studies (2), we found strong evidence that cell surface APLP2 is routed to lysosomes following endocytosis. Total cellular APLP2 showed a significant degree of colocalization with lysosomes in HepG2 cells that was not altered by the presence of PCSK9 or PCSK9/J16 (supplemental Fig. 5, A and B). In addition, an anti-APLP2 monoclonal antibody exogenously added to HepG2 cells was internalized and highly colocalized with lysosomes (Fig. 7*B*).

When PCSK9-488 was added in combination with the anti-APLP2 antibody and allowed to internalize, the two proteins were almost completely colocalized with an average icorr value of 0.84 ± 0.09 (Fig. 7*C*). Importantly, PCSK9/APLP2-positive puncta were frequently colocalized with lysosomes (Fig. 7*C*, *arrows*). Thus, PCSK9 and APLP2 are trafficked along the same endocytic route to lysosomes. In contrast, APP and internalized PCSK9 were not significantly colocalized (Fig. 8*B*), providing additional evidence that APP does not play a direct role in PCSK9 trafficking.

##### PCSK9 Induces Trimeric Complex Formation with LDLR and APLP2 at Endosomal pH

During our studies, we noted that LDLR and APLP2 colocalized somewhat throughout the cell with an icorr value of 0.60 ± 0.04 (Fig. 7*D*). Exogenously added PCSK9 significantly enhanced the degree to which LDLR and APLP2 were colocalized, resulting in almost complete colocalization between the two proteins with an icorr value of 0.86 ± 0.08 (Fig. 7*D*). Importantly, this effect could be reversed by the presence of J16 (Fig. 7*D*; icorr value of 0.54 ± 0.08), indicating that PCSK9/LDLR interactions are required for the observed PCSK9-mediated LDLR/APLP2 cellular colocalization.

**FIGURE 9.**
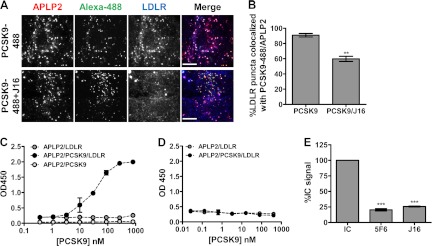
**Recombinant PCSK9, APLP2, and LDLR form a trimeric complex *in vitro*.** *A*, LDLR (*blue*) colocalization with PCSK9-488 (*green*) and APLP2 (*red*) in the absence or presence of J16 in HepG2 cells as indicated. *Scale bars*, 10 μm. *B*, quantification of *A* as the percentage of LDLR puncta colocalized with puncta positive for both PCSK9-488 and APLP2. Shown is the average with S.E. (*error bars*) from three independent experiments. *C*, ELISA in pH 6.0 buffer of LDLR-ECD association with APLP2-ECD with increasing concentrations of PCSK9. Shown is the average with S.D. (*error bars*) of triplicates from a representative experiment. *D*, same as *C* but performed at pH 7.4. *E*, ELISA of APLP2-PCSK9-LDLR complex with 4 μg/ml PCSK9 and 12 μg/ml IC, J16, or 5F6. Shown is the average with S.E. (*error bars*) from three independent experiments. **, *p* = 0.0016; ***, *p* ≤ 0.0005.

To determine whether PCSK9 localized to the same intracellular compartments as LDLR and APLP2, PCSK9-488 was added to HepG2 cells, and cells were stained for APLP2 and LDLR. All three proteins were highly colocalized, suggesting that they are trafficked together (Fig. 9*A*). Consistent with our previous finding that J16 does not alter PCSK9 trafficking, APLP2 and PCSK9-488 colocalization was not disrupted by the presence of J16, whereas LDLR dramatically diverged away from PCSK9-488/APLP2-positive puncta (Fig. 9*A*). Indeed, the percentage of LDLR puncta associated with PCSK9-488/APLP2 was ∼90% with IC and only 60% with J16 (Fig. 9*B*). These data support the notion that PCSK9 may enhance the lysosomal targeting of LDLR by bridging it to APLP2.

To explore this possibility, we next tested whether recombinant PCSK9 can physically bridge LDLR-ECD to APLP2-ECD. Premixing with PCSK9 greatly and dose-dependently enhanced the binding of LDLR-ECD to an ELISA plate coated with APLP2-ECD at pH 6.0 (Fig. 9*C*) but not at pH 7.4 (Fig. 9*D*). Importantly, assembly of this triplex could be blocked by J16 or 5F6 (Fig. 9*E*), indicating that both PCSK9/APLP2 and PCSK9/LDLR interactions are required for bridging LDLR to APLP2.

##### APLP2 Is Essential for PCSK9 Trafficking to Lysosomes

**FIGURE 10.**
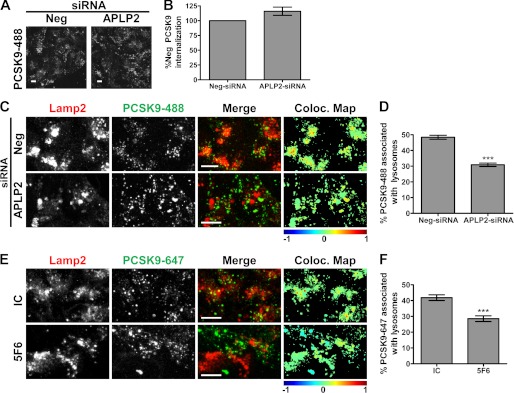
**Effects of disruption of PCSK9/APLP2 interaction on PCSK9 trafficking.** *A*, PCSK9-488 internalization in negative control siRNA (*Neg*) or APLP2 siRNA knockdown cells. *B*, quantification of *A* shown as the average percent PCSK9 intensity of the negative control with S.E. (*error bars*) from three independent experiments. *C*, PCSK9-488 (*green*) colocalization with Lamp2 (*red*) in negative control siRNA (*Neg*) or APLP2 siRNA-treated HepG2 cells. The colocalization (*Coloc.*) color map on the *far right* shows the intensity of colocalization between PCSK9-488 and Lamp2 as indicated by the *color bar* below. In these color maps, the −1 to +1 heat map depicts the measured icorr values. Negatively correlated relationships (icorr values between −1 and 0) are shown in *blue-green* colors, whereas positive correlations (icorr values between 0 and 1) are represented by warmer *yellow-red* colors. *Scale bars*, 10 μm. *D*, quantification of *C* shown as the average percentage of PCSK9 puncta colocalized with Lamp2 with S.E. (*error bars*) from three independent experiments. *E*, colocalization of PCSK9-488 (*green*) combined with IC or 5F6 with Lamp2 (*red*) in HepG2 cells. Colocalization color maps on the *far right* show the intensity of colocalization between PCSK9-488 and Lamp2 as indicated by the *color bar* below and as described in *C. Scale bars*, 10 μm. *F*, quantification of *E* shown as the average percentage of PCSK9 puncta colocalized with Lamp2 with S.E. (*error bars*) from three independent experiments. ***, *p* < 0.001.

Recent studies have shown that the CHRD of PCSK9 is involved in its postendocytic sorting, and it has been hypothesized that this occurs via interactions with unknown proteins (19, 20). We observed that PCSK9-488 internalization was not altered in APLP2 siRNA knockdown cells relative to negative control siRNA-treated cells (Fig. 10, *A* and *B*). Thus, the effect of APLP2 knockdown on PCSK9 function was not due to a loss of internalization; however, consistent with the role of the CHRD, it may indicate a role in postendocytic sorting.

To test whether APLP2 is necessary for PCSK9 trafficking to lysosomes, we added PCSK9-488 to APLP2 siRNA-treated cells and determined its lysosomal trafficking efficiency. The percentage of PCSK9-488 localized with lysosomes was reduced to 30.9 ± 0.62% in APLP2 knockdown cells from 48.4 ± 0.72% observed in negative control cells (Fig. 10, *C* and *D*). These results are also evident in the colocalization color maps of PCSK9-488 and Lamp2 where the increased presence of *yellow*/*red* in the negative siRNA cells relative to the cooler colors in the APLP2 siRNA cells illustrates that postendocytic PCSK9 lysosomal trafficking is shifted away from lysosomes when APLP2 levels are diminished (Fig. 10*C*, *far right column*). Consistently, PCSK9-mediated LDLR trafficking to lysosomes was also significantly reduced in APLP2 siRNA-treated cells (31.8 ± 0.93%) as compared with the negative control cells (43.3 ± 1.12%; supplemental Fig. 5, C and D). Moreover, the presence of 5F6 significantly reduced the amount of PCSK9-647 that reached lysosomes (Fig. 10, *E* and *F*). Together, these data indicate that PCSK9/APLP2 interactions are important for postendocytic PCSK9 trafficking to lysosomes.

## DISCUSSION

We previously identified and engineered an anti-PCSK9 antibody, J16, that completely blocks PCSK9 binding to LDLR (24). Surprisingly, our studies indicated that J16 is degraded in a PCSK9-dependent manner, suggesting that PCSK9 is internalized and trafficked to lysosomes even when its direct interaction with LDLR is blocked (25).

In this study, we confirmed that J16 does not alter PCSK9 trafficking; rather, exogenously added PCSK9 bound to J16 is still endocytosed and routed to lysosomes. Importantly, our study was done without overexpression and therefore allowed us to characterize PCSK9 interactions and trafficking under more relevant physiological conditions. The reported blocking of PCSK9 internalization by EGF-AB peptide that seemingly contradicts our observations (17) could be due to direct binding of EGF-AB peptides to other LDLR-interacting proteins that in turn could affect processes such as receptor clustering that may be required for PCSK9 endocytosis. Consistent with this idea, we found that siRNA knockdown of LDLR impairs PCSK9 endocytosis even when PCSK9 is bound to J16 or when PCSK9 is mutated and cannot directly bind LDLR. We therefore hypothesize that LDLR may regulate PCSK9 endocytosis by affecting endocytic adaptor recruitment or receptor clustering. In support of this, the presence of LDLR is required for recruitment of autosomal recessive hypercholesterolemia (ARH), to the plasma membrane (36), and it has been shown in previous studies that PCSK9 endocytosis in hepatic cells relies on this endocytic adaptor protein (37, 38).

Following endocytosis, transmembrane proteins are generally sorted to various cellular compartments according to signals found in their cytosolic tails (4). Endocytosed LDLR predominantly follows the default recycling pathway back to the cell surface, so the means by which PCSK9 diverts LDLR to lysosomes remains an important question. A recent, exhaustive study showed that PCSK9 does not follow a canonical lysosomal trafficking route (39), whereas other studies reported that the cytosolic tail of LDLR is not necessary for PCSK9 targeting of LDLR to lysosomes (11, 40). Moreover, PCSK9 promotes lysosomal degradation of other receptors in the LDLR family such as ApoER2 and VLDL receptor as well as the β-secretase BACE1 and our neutralizing anti-PCSK9 antibody. Thus, it is intriguing to postulate that PCSK9 mediates lysosomal transport and subsequent degradation of its targets via a general mechanism from the luminal side of the membrane perhaps via interactions with a second transmembrane partner.

In direct support of this idea, a recent study showed that the M2 module of the CHRD of PCSK9 is required for PCSK9 to degrade LDLR via its endocytic pathway. Importantly, the authors found that this domain is not essential for endocytosis of PCSK9, and they therefore hypothesized that the M2 module of the CHRD interacts with another, unknown protein to mediate postendocytic sorting of PCSK9 (20). Interestingly, the authors of this study also noted that the M2 module is not required for the intracellular route by which PCSK9 functions. Given the divergent structural requirements of PCSK9, the two routes may therefore be able to regulate or compensate for each other. For instance, if PCSK9 bound to LDLR is endocytosed but unable to be routed to lysosomes, it may intersect with components of the intracellular route to carry out this function.

In this study, we sought to identify PCSK9 binding partners that may be involved in postendocytic sorting of PCSK9. In doing so, we discovered that PCSK9 interacts via its CHRD with both APP and its close family member, APLP2, in a pH-dependent manner. Interestingly, APLP2 has been shown previously to transport other transmembrane proteins, specifically MHC class I molecules, to lysosomes (2, 41), whereas APP has been previously linked to the LDLR-related protein family in a variety of studies (42, 43). Moreover, PCSK9 has been shown to regulate levels of BACE1, one of the enzymes that cleaves APP to form the Aβ peptide associated with Alzheimer disease (44).

Interestingly, inhibiting PCSK9 interactions with APP or APLP2 with the anti-PCSK9 monoclonal antibody 5F6 neutralized PCSK9-mediated LDLR degradation. While dissecting the individual roles of APP and APLP2 in PCSK9 function, we found that knockdown of APLP2, but not APP, resulted in significant loss of PCSK9-mediated degradation of LDLR, directly implicating APLP2 in PCSK9 function. Moreover, the loss of APLP2 or presence of 5F6 significantly shifted postendocytic PCSK9 trafficking away from lysosomes. These data, combined with the observed APLP2 interaction with the CHRD of PCSK9 at acidic endosomal pH, led us to hypothesize that APLP2 is directly involved in the postendocytic lysosomal transport of PCSK9.

Importantly, PCSK9, APLP2, and LDLR are likely endocytosed in the same compartments because exogenously added PCSK9 localizes to LDLR/APLP2-positive clusters on the cell surface despite an inability to directly interact with APLP2 at neutral pH. This could be due to binding to an as of yet unknown PCSK9-binding membrane protein that is also internalized in APLP2 and LDLR endosomes or through some cofactor that facilitates binding between PCSK9 and APLP2 or LDLR at neutral pH. Following internalization, exposure to endosomal pH would then enable PCSK9 to bind to APLP2. Indeed, PCSK9 and APLP2 are almost always colocalized following internalization, and PCSK9 follows the same endocytic route as APLP2 from the cell surface to lysosomes. APLP2 follows this endolysosomal route regardless of the presence of PCSK9, and PCSK9 does not mediate APLP2 degradation; we therefore hypothesize that cellular APLP2 protein levels are regulated and maintained in part via this endolysosomal route. Interestingly, a significant amount of APLP2 and LDLR clusters to the same regions on the cell surface in the absence of PCSK9 although to a lesser degree, indicating that the two transmembrane proteins may normally be internalized together. It is therefore possible that APLP2 is involved in basal LDLR turnover and that PCSK9 functions by harnessing this pathway.

Following endocytosis, we hypothesized that PCSK9 may facilitate complex formation between the three proteins in endosomes. Indeed, PCSK9 can physically bridge recombinant LDLR-ECD to APLP2-ECD at endosomal pH. Supporting that this trimeric complex exists in cells, addition of PCSK9 dramatically alters LDLR cellular localization toward APLP2, and this effect can be reversed by J16. In contrast, PCSK9 can be shifted away from its lysosomal route by the presence of 5F6 or a reduction of APLP2, indicating that PCSK9 trafficking is dependent on its interaction with APLP2. Together, these data support a model in which APLP2 binds to the CHRD of PCSK9 in endosomes and transports PCSK9 while bound to its targets (*e.g.* LDLR or J16) to lysosomes. Interestingly, these conclusions complement those made by Saavedra *et al.* (20) regarding mediation of postendocytic sorting of PCSK9 by its CHRD.

In contrast to APLP2, we observed an increased sensitivity to PCSK9 in APP knockdown cells. This in combination with a lack of cellular colocalization between PCSK9 and APP led us to conclude that APP does not actively regulate PCSK9 function but instead may play a more passive, protective role perhaps by competing with APLP2 for PCSK9 binding. Moreover, knockdown of APP significantly diminished LDLR mRNA levels. This finding is consistent with published studies showing that APP protein levels are directly proportional to LDLR mRNA levels (43). Together, our data provide additional links between the LDLR-related protein family and the amyloid precursor protein family while demonstrating that APP and APLP2 differentially regulate LDLR levels.

In this study, we discovered a novel, pH-dependent interaction between PCSK9 and APLP2 that facilitates PCSK9 lysosomal delivery and function. Based on our observations, we propose that PCSK9 is involved in a novel lysosomal transport complex that would allow it to degrade multiple targets, including anti-PCSK9 blocking antibodies, by the same mechanism. Importantly, these findings are of significance for PCSK9 antagonist therapeutic programs and may provide an alternative avenue by which PCSK9 function can be attenuated. Furthermore, this proposed mechanism allows for a soluble messenger from either a local or distant source to mediate what has previously been considered a cell-autonomous process.

## Supplementary Material

Supplemental Data

## Footnotes

2 The abbreviations used are:

APLP2: amyloid precursor-like protein 2
IC: isotype control
Lamp2: lysosome-associated membrane protein 2
LDLR: low density lipoprotein receptor
PCSK9: proprotein convertase subtilisin/kexin type 9
TFNR: transferrin receptor
APP: amyloid precursor protein
CHRD: Cys-His-rich domain
ECD: extracellular domain
LPDS: *lipoprotein-deficient serum*
Bis-Tris: 2-[bis(2-hydroxyethyl)amino]-2-(hydroxymethyl)propane-1,3-diol
icorr: correlation index
IP: immunoprecipitation.
