# Supplementary material for: Characterization of Proprotein Convertase Subtilisin/Kexin Type 9 (PCSK9) Trafficking Reveals a Novel Lysosomal Targeting Mechanism via Amyloid Precursor-like Protein 2 (APLP2)
Source: J Biol Chem. 2013 Feb 19;288(15):10805–18. doi: 10.1074/jbc.M113.453373 (PMC3624461; doi:10.1074/jbc.M113.453373)
Supplement: Supplemental Data [file supp_288_15_10805__index.html]

Characterization of Proprotein Convertase Subtilisin/Kexin Type 9 (PCSK9) Trafficking Reveals a Novel Lysosomal Targeting Mechanism via Amyloid Precursor-like Protein 2 (APLP2) — PCSK9 Functions via a Lysosomal Transport Complex — Supplemental Data 

# Characterization of Proprotein Convertase Subtilisin/Kexin Type 9 (PCSK9) Trafficking Reveals a Novel Lysosomal Targeting Mechanism via Amyloid Precursor-like Protein 2 (APLP2)

## Supplemental Data

**Files in this Data Supplement:**

- Supplemental figures and legends (.pdf, 2.8 MB) - 5 Supplemental figures and their legends.
