# Supplementary material for: Characterization of Proprotein Convertase Subtilisin/Kexin Type 9 (PCSK9) Trafficking Reveals a Novel Lysosomal Targeting Mechanism via Amyloid Precursor-like Protein 2 (APLP2)
Source: J Biol Chem. 2013 Feb 19;288(15):10805–18. doi: 10.1074/jbc.M113.453373 (PMC3624461; doi:10.1074/jbc.M113.453373)
Supplement: Supplemental Data [file supp_M113.453373_jbc.M113.453373-1.pdf]

SUPPLEMENTAL DATA

Supplemental Figure 1. PCSK9 localization relative to ER or Golgi. (A) Exogenously added PCSK9-488 (green) in HepG2 cells costained with Calnexin (ER marker; red). (B) Exogenously added PCSK9-488 (green) in HepG2 cells costained with Golgin 97 (Golgi marker; red). Scale bars, 10μm.

Supplemental Figure 2. Characterization of siRNA knockdowns. (A) Western blot of LDLR and transferrin receptor (TFNR) from negative siRNA (neg) or LDLR siRNA treated HepG2 cells. Quantifications shown on the right were normalized to TFNR signal and shown as average percent LDLR signal of negative siRNA treated cells, with SEM from 3 independent experiments. (B) Same as (A), except cells were treated with APLP2 siRNA and membranes were probed for APLP2. (C) Same as (A), except cells were treated with APP siRNA and membranes were probed for APP. (D) qPCR of LDLR mRNA levels in Neg, APLP2, LDLR, or APP siRNA knockdown cells. Quantified as described in methods; shown as average fold change over negative control with SEM from 3 independent experiments.

Supplemental Figure 3. LDLR surface expression and LDL internalization in siRNA treated cells. (A) Surface expressed LDLR (green) and DAPI staining (blue) in negative (Neg), APLP2, APP, or LDLR siRNA treated HepG2 cells. Scale bars, 10μm. (B) Confocal microscopy of internalized LDL-BODIPY (LDL) in negative (Neg), APLP2, APP, or LDLR siRNA treated HepG2 cells. Scale bars, 10μm.

Supplemental Figure 4. PCSK9 cell surface localization relative to LDLR and LRP8 in HepG2 cells. (A) PCSK9-647 (red) colocalization with cell surface LRP8 (green) in the presence of isotype control (IC) or J10 (blue). (B) PCSK9-647 (red) colocalization with cell surface LDLR (green) in the presence of IC or J10 (blue). Scale bars, 10μm. (C) Percent PCSK9 puncta associated with LDLR or LRP8 puncta in the presence of IC or J16. Shown as average with SD from at least 500 PCSK9-647 puncta.

Supplemental Figure 5. APLP2 lysosomal trafficking in HepG2 cells. (A) APLP2 (green) colocalization with Lamp2 (red) in the absence or presence of PCSK9, or PCSK9/J16 complex as indicated. Colocalization colormap is shown on the far right. Scale bars, 10μm. (B) Quantification of (A) shown as average percent APLP2 icorr values in PCSK9 or PCSK9/J16 treated cells relative to untreated cells. Shown with SEM from 3 independent experiments. (C) LDLR (green) colocalization with Lamp2 (red) in the presence of PCSK9 in negative control (neg) or APLP2 siRNA knockdown cells as indicated. Scale bars, 10μm. (D) Quantification of (C) showing average percent LDLR puncta associated with Lamp2 puncta, with SEM, from 3 independent experiments.

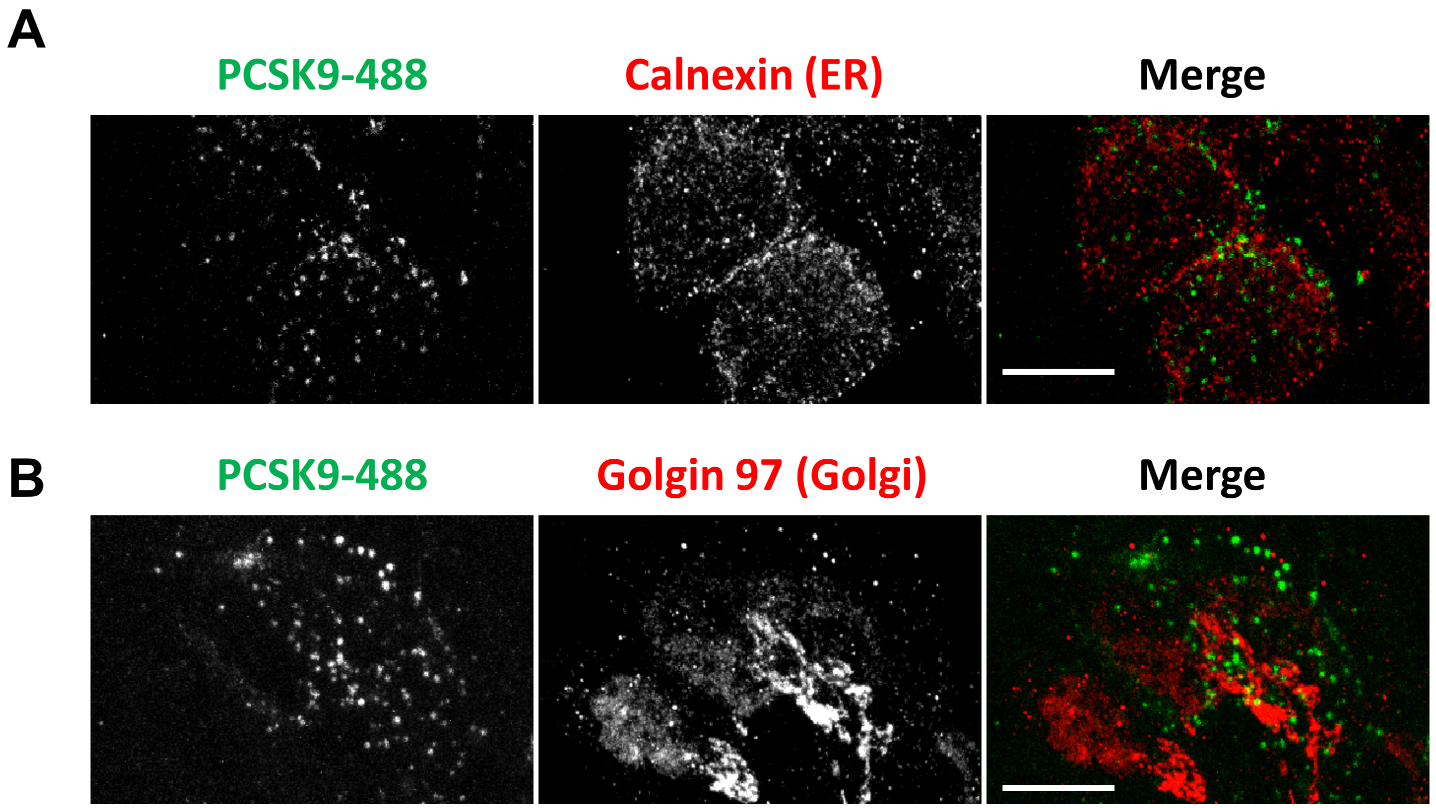

Supplemental Figure 1

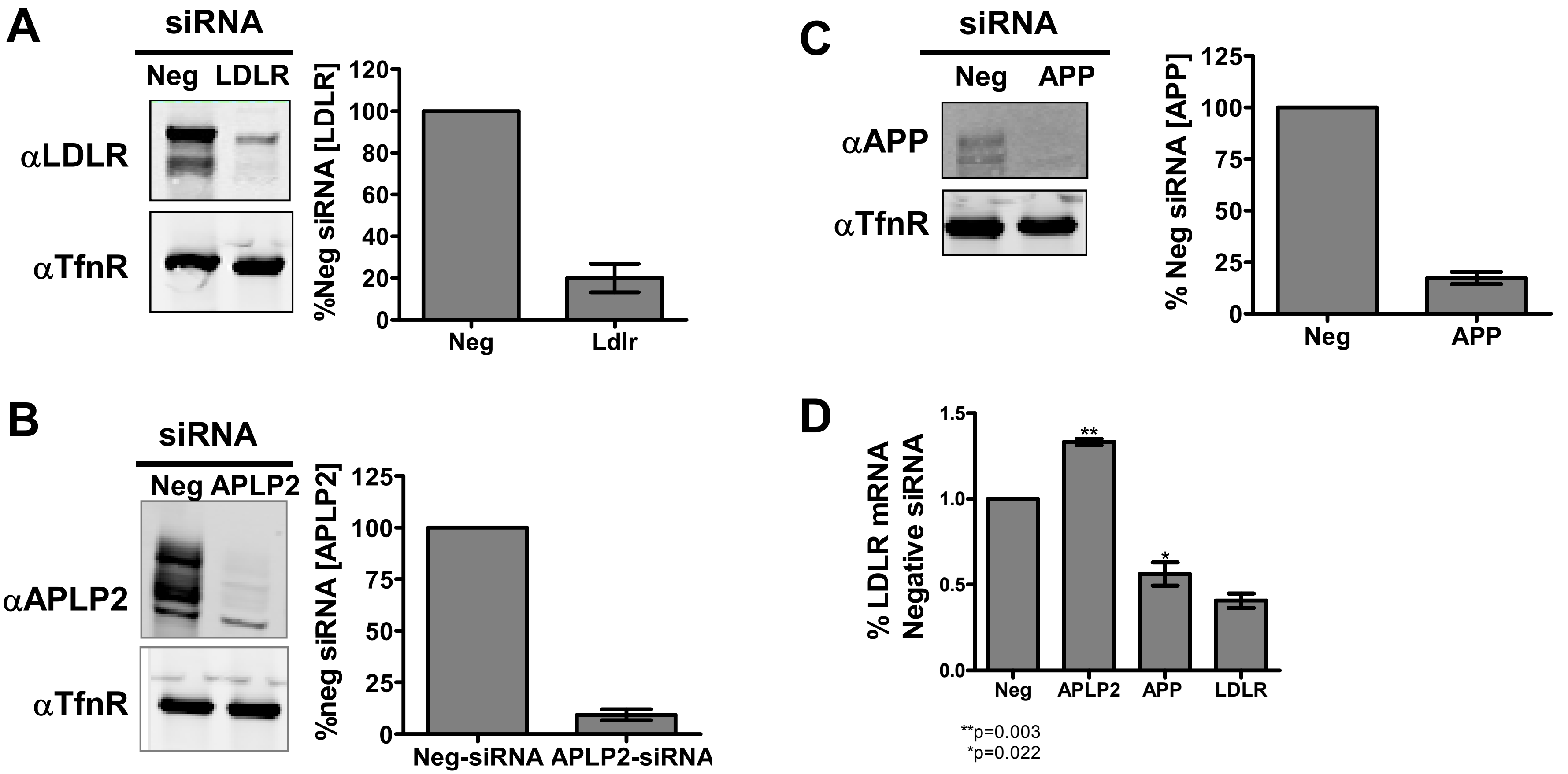

Supplemental Figure 2

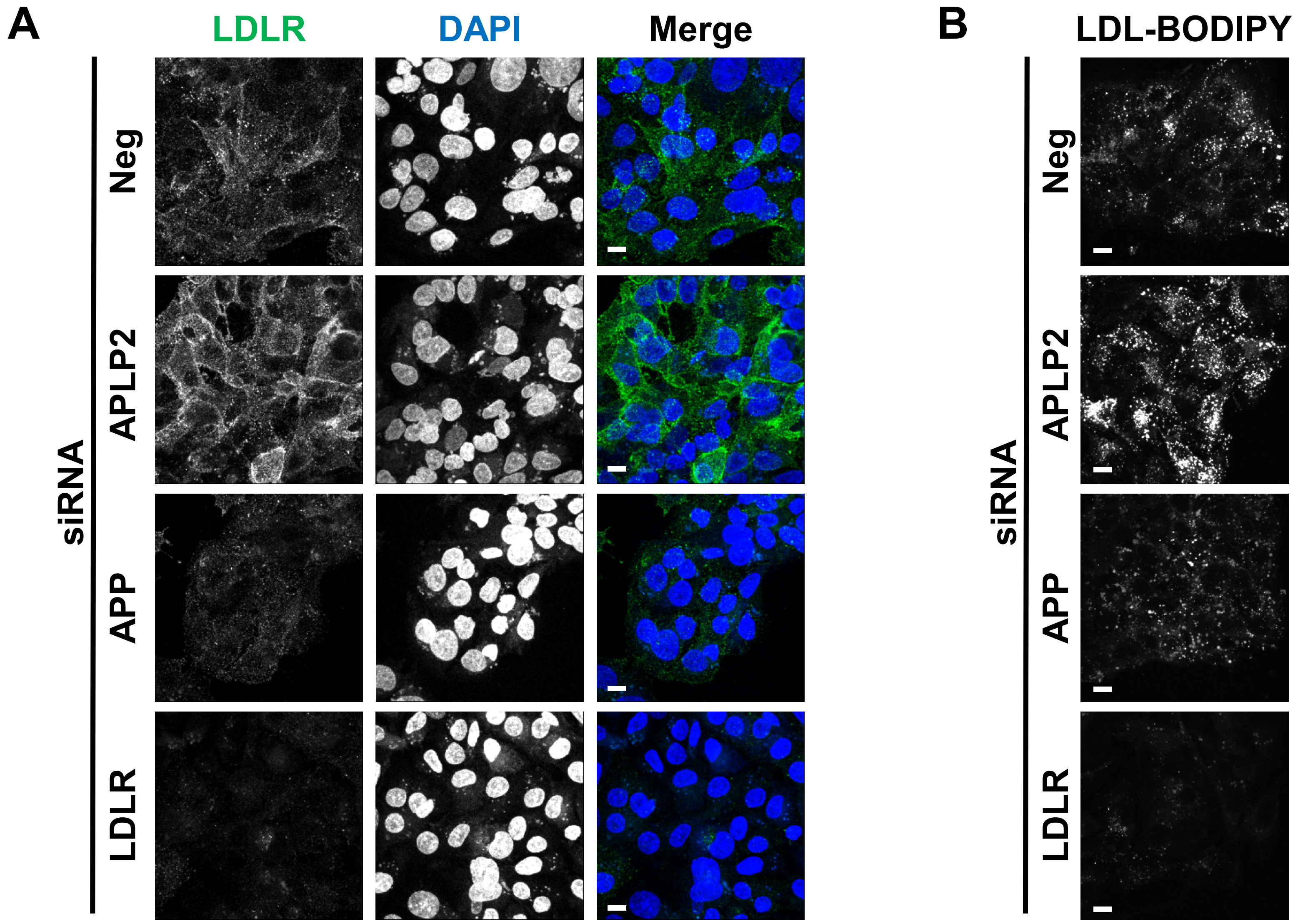

Supplemental Figure 3

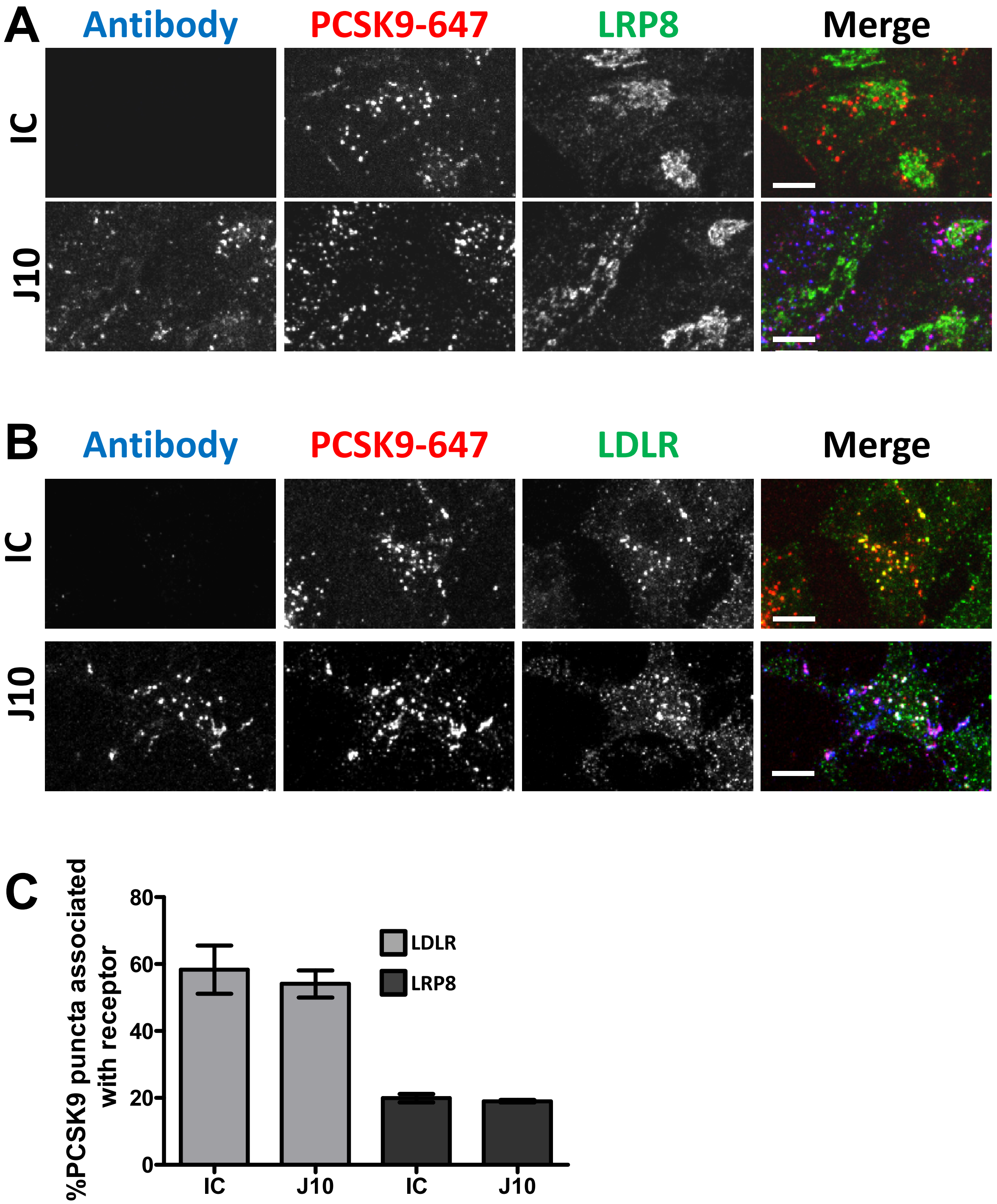

Supplemental Figure 4

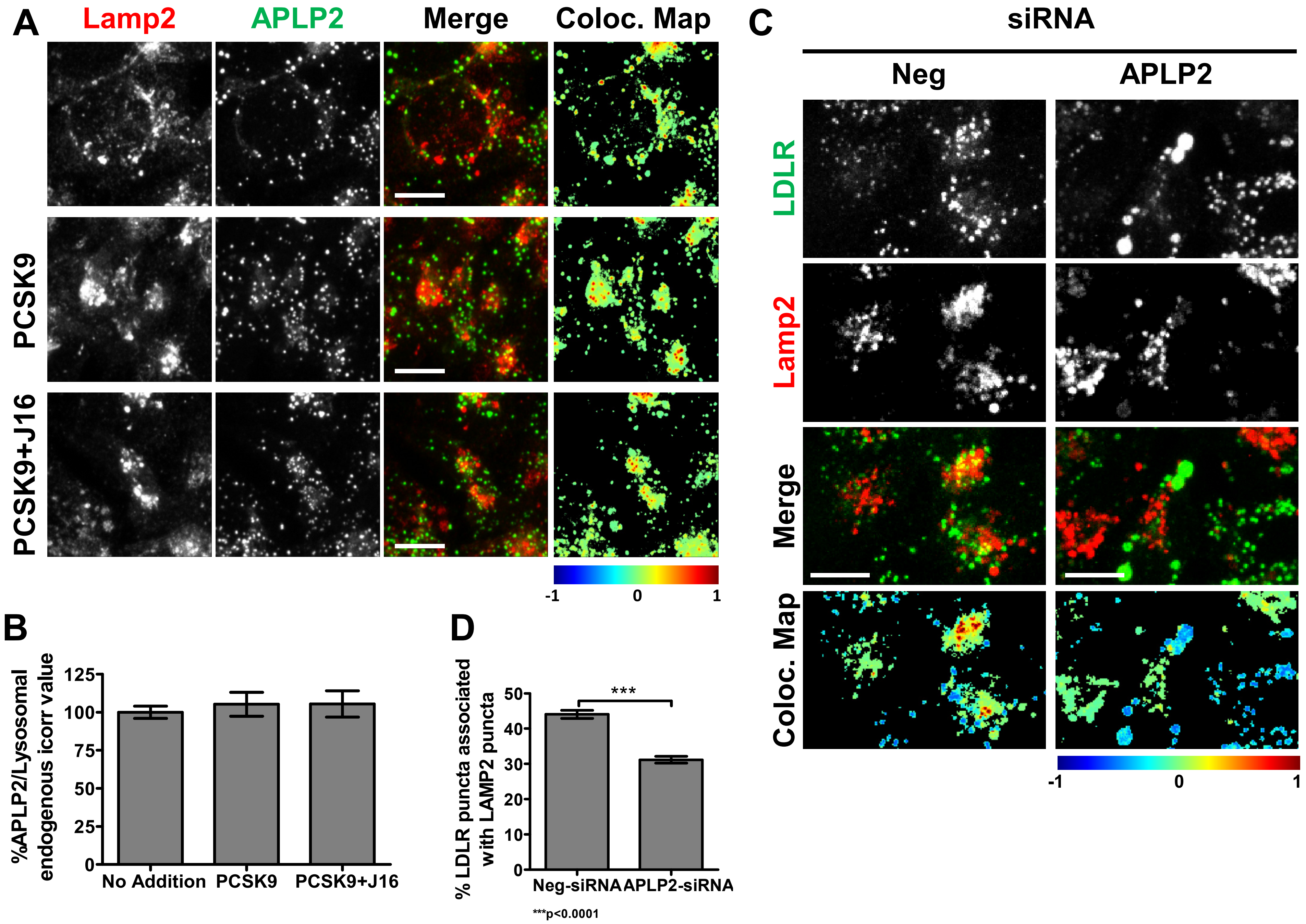

Supplemental Figure 5
